# Skin Autofluorescence Mirrors Surrogate Parameters of Vascular Aging: An Enable Study

**DOI:** 10.3390/nu15071597

**Published:** 2023-03-25

**Authors:** Tianxing Du, Beate Brandl, Hans Hauner, Thomas Skurk

**Affiliations:** 1 ZIEL—Institute for Food & Health, Technical University of Munich, 85354 Freising, Germany; 2 Clinical Nutritional Medicine, Else Kroener-Fresenius-Centre of Nutritional Medicine, School of Life Sciences, Technical University of Munich, 85354 Freising, Germany; 3 Institute of Nutritional Medicine, School of Medicine, Klinikum Rechts der Isar, Technical University of Munich, 80333 Munich, Germany

**Keywords:** skin autofluorescence, advanced glycation end-products, vascular health, atherosclerosis risk, intima–media thickness, pulse wave velocity

## Abstract

Advanced glycation end-products (AGEs) are implicated in vascular aging due to their pro-inflammatory properties. Skin autofluorescence (SAF) is a measure to estimate their deposition. It is an easily quantifiable marker that has been shown to correlate with cardiovascular risk and parameters of metabolic diseases. Herein, we compared skin autofluorescence with other techniques indicating increased cardiovascular diseases, namely, pulse wave velocity (PWVao) and intima–media thickness (IMT). We also studied the impacts of other parameters in deeply phenotyped cohorts of young, middle-aged, and older individuals. SAF, aortic PWVao, and IMT proved to be significantly correlated with each other and with age. However, based on a moderator analysis, we could not show that these associations were affected by age. Some specific parameters such as creatinine and CRP were found to be significantly associated with skin AGE values after adjusting for confounding variables. In conclusion, SAF is a simple screening tool for vascular health with comparable power to more elaborated technical tests.

## 1. Introduction

Advanced glycation end-products (AGEs) are a group of glycated proteins or lipids that have attracted significant interest in recent years due to their established role in the aging process and their involvement in the development of multiple chronic diseases. Through a process called the Maillard reaction, AGEs are formed between reducing sugars and proteins, lipids, or nucleic acids, resulting in an irreversible crosslinking of molecules. AGEs can be produced endogenously in the body or ingested via different foods. Once taken up, they are resistant to proteolytic degradation and, therefore, very stable if deposited in tissues. AGEs signal through a 35 kDa receptor for advanced glycation end-products (RAGE, also called AGRE) from the immunoglobulin superfamily involving nuclear factor kappa B (NF-κB)-activation and, thus, mediate inflammatory signals. According to their pro-inflammatory character, AGEs are implicated in vascular aging, and, therefore, studies have linked AGEs to the development of cardiovascular disease [1], diabetes [2], osteoporosis [3], and neurodegenerative diseases [4,5], which are considered to grow in an inflammatory background.

One of the ways that AGEs accumulate in the body is through crosslinking with proteins such as collagen, leading to the formation of long-living proteins, which can also be monitored in the skin [6]. Because of the significant involvement of AGEs in the aging process, they are considered as potential surrogates to indicate general health. Facilitated access to a reliable measure of AGE deposition might also have therapeutic implications. AGE crosslink-breakers are a newly discovered class of therapeutics that may be potentially valuable for the prevention or treatment of chronic cardiovascular diseases [7].

AGEs mostly appear with a brown color. As such, it is possible to detect Maillard products via skin autofluorescence (SAF) according to their physical properties. This is one of the most promising ways to assess the abundance of AGEs non-invasively [8,9]. However, it is reported that SAF measurement can be influenced by multiple factors such as the concentrations of hemoglobin in skin capillaries and other dermal factors such as melanin pigmentation, blood flow, renal function, or cosmetics [10,11]. Despite these limitations, SAF measurement remains a valuable tool for assessing AGEs in the skin.

In addition to SAF measurement, there are already other established surrogate parameters, namely, aortic pulse wave velocity (PWVao) and intima–media thickness (IMT). Meanwhile, PWVao reflects a composite measure of the different elasticities of large arteries to indicate their ability for vascular deformation in response to pressure, and IMT measures the inner parts of an artery, typically the carotid artery, namely, the intima and the media. Both techniques were shown to reflect changes associated with human aging [12,13,14] and, in particular, have been confirmed to indicate aging-related complications such as cardiovascular diseases and type 2 diabetes [15,16,17,18,19]. Besides such age-related metabolic disorders, it is worth noting that AGE levels in skin biopsies in other previous studies have, furthermore, predicted microvascular complications in type 1 diabetes [20,21].

As SFA, the other two techniques to measure PWVao and IMT, either using ultrasound imaging or oscillometric methods, respectively, are non-invasive, relatively inexpensive, and reproducible. However, these methods differ in their methodological complexities. For this reason, the aim of our study was to analyze (1) how age interferes with different measures of PWVao, skin SAF values, and IMT, and (2) how these three parameters are interrelated. (3) We, furthermore, identified extrinsic markers for skin aging with a focus on selected phenotypic factors in adolescent and adult cohorts of our nutrition cluster enable. Our results may help to improve our understanding of the aging process and develop new strategies for the prevention and treatment of age-related diseases.

## 2. Methods

### 2.1. Ethics Statement

The ethics committee of the Technical University of Munich approved the study protocol (no. 425/15 S and no. 201/17 S) to ensure that this research was conducted ethically and in compliance with all relevant regulations and guidelines. The committee evaluated the study design, including the recruitment of participants, data collection methods, and potential risks and benefits to the participants.

To ensure transparency and accountability, this study was registered in the German Register of Clinical Trials (DRKS00009797). By registering this study, we made the study design and protocol publicly available, which increases the scientific rigor and reproducibility of the research process.

As a crucial aspect of ethical research, written informed consent was obtained from all subjects involved in this study. We provided a detailed explanation of the research procedures, potential risks and benefits, and the participant’s right to withdraw from the study at any time. Participants were also informed about the confidentiality of their personal information and data protection regulations, and their consent was documented in writing. All personal data collected during this study were anonymized to prevent any unintended disclosure of participants’ identities. We also followed strict protocols for data storage, handling, and sharing to ensure that participants’ personal information was safeguarded and only accessible to authorized members of the research team.

### 2.2. Study Design

Data were collected in the *enable* study center at the ZIEL Institute for Food and Health, Freising, Germany. This study was performed in the Munich area from March 2016 to February 2018. The original cohorts of various age ranges within the competence cluster of the nutrition research *enable* have been described elsewhere [22].

For the analysis of SAF and PWVao, we used a study population consisting of 3 groups of individuals: adolescents (from 18 to 25 years), middle-agers (from 40 to 65 years), and older adults (from 75 to 85 years). A total of 329 individuals were recruited for the study, with 93 adolescents, 187 middle-agers, and 49 older adults. After removing volunteers lacking sufficient data, 309 subjects remained in this group. Volunteers were excluded from this study if they had a history of smoking; known diabetes mellitus type 1 or 2; severe diseases such as coronary heart disease, chronic liver affections, or kidney diseases; and immobility. For our comprehensive examination protocol, participants were required to fast for 12 h overnight.

To additionally investigate IMT, we took advantage of a subgroup of the middle-aged population in the group of 40–65-year-old participants who were recruited for an interventional nutrition study [23]. All enrolled individuals were of Caucasian descent. Besides anthropometric parameters such as height, weight, body composition measured via bioimpedance analysis, and hip and waist circumference, only subjects with a complete data set from PWVao, IMT using ultrasound, and SAF were included in this further analysis, resulting in a final sample size of 77 individuals.

### 2.3. Data Collection

#### 2.3.1. Anthropometric Measurements

Anthropometric measurements are an essential component of clinical research as they provide a quantitative assessment of body size and composition. In this study, all anthropometric parameters and body composition, including waist-to-hip ratio (WHR) and body mass index (BMI), were measured in the morning with the Seca 213 Stadiometer device (Seca GmbH & Co KG, Hamburg, Germany). To further ensure accuracy, established standard operating procedures (SOPs) were followed during all measurements. Additionally, all participants were strictly required to have fasted overnight before the anthropometric experiments.

#### 2.3.2. Blood Samples

Venous blood samples were obtained by medical doctors from fasted participants. Lipid parameters such as total cholesterol, triglycerides, high-sensitivity C-reactive protein (hsCRP), and insulin were analyzed by a certified laboratory (Synlab Analytics & Service Germany GMBH; Munich, Germany). Blood glucose concentrations were determined using HemoCue Glucose 201+ (plasma-calibrated, HITADO GmbH, Möhnesee, Germany). Glomerular filtration rate (GFR) was calculated as mL/min/1.73 m^2^ from serum creatinine using the Chronic Kidney Disease Epidemiology Collaboration (CKD-EPI) equation.

#### 2.3.3. Pulse Wave Velocity of the Aorta (PWVao)

The TensioMed Arteriograph device TL 2 (TensioMed Ltd., Budapest, Hungary) was used to measure PWVao. Strict instructions were given to participants prior to the measurements. Participants were asked to refrain from eating, drinking coffee, and smoking three hours before the test. They were instructed to avoid drinking alcohol 10 h before the measurements as well. Before the measurements, the participants rested for at least 10 to 15 min in a quiet room at normal room temperature.

#### 2.3.4. Skin SAF Measurement

Skin SAF was measured using the AGE Reader (AGE Reader mu, Type DMU00100, Diagn-Optics; Groningen, The Netherlands). The rubber cushion-like top of the device was designed to hold the volar side of a subject’s lower forearm and seal the glass measuring window from any disturbing light. Each participant’s elbow was in line with the edge of the cushion. The skin was free of tattoos, skin creams, birthmarks, or scars in the measured area. Each experiment was performed on the right arms of the participants. The whole process took approximately 12 s. The data were immediately available and recorded.

#### 2.3.5. Intima–Media Thickness (IMT)

In order to assess the thickness of the common carotid artery, the IMT was measured with the high-frequency ultrasound ACUSON X700 device (Siemens Healthcare GmbH, Erlangen, Germany). Participants were placed in a supine position for the measurements. The IMT was determined during the peak systole according to a parallel ECG reading. Images were taken, centered approximately 10 mm below the carotid artery bulb.

#### 2.3.6. Oral Glucose Tolerance Test (OGTT)

To determine fasting blood glucose and insulin levels, blood samples were taken prior to the ingestion of a solution containing 75 g α-D(+)-Glucose (water-free) in 300 mL of tap water. Further blood glucose measurements were performed at 30, 60, 90, 120, 180, and 240 min. The area under the curve (AUC) of the OGTT was calculated using the trapezoid method.

#### 2.3.7. Questionnaires

In addition to the objective measures above, participants had to fill out questionnaires on physical activity, dietary intake, and general health, as recently described in [21]. The physical activity levels (exercise frequency) of participants were self-reported using 6 categories from level 1 (“once a day”) to level 6 (“never”).

#### 2.3.8. Data Analysis

The dataset was first set up and edited with the spreadsheet application Excel (Microsoft, Version 16.58). Data analysis was conducted in a programming environment of Python version 3.9.0 [24].

Notably, the multiple linear regression model was established using statsmodel version 0.13.2 [25]. The Spearman’s correlation coefficient was calculated using Pingouin version 0.5.1 [26]. Categorical data were numerically labeled using the *LabelEncoder* function in sklearn version 1.0.2 [27]. Quentin André’s PyProcessMacro version 1.0.12, a transforming Python package from Process Macro, was used to analyze the potential moderating effects of age between skin SAF and IMT and PWVao [28].

The residuals of both cohorts’ data were tested for their normality using the Shapiro–Wilk test. *p*-values were determined as follows: not significant (n.s.): *p* ≤ 1.00; *: 1.00 × 10^−2^ < *p* ≤ 5.00 × 10^−2^; **: 1.00 × 10^−3^ < *p* ≤ 1.00 × 10^−2^; ***: 1.00 × 10^−4^ < *p* ≤ 1.00 × 10^−3^; and ****: *p* ≤ 1.00 × 10^−4^. The significant moderation effect of age was verified based on the values of its confidence intervals ULCI (upper level of confidence interval) and LLCI (lower level of confidence interval).

In the regression analysis, the consideration of the variance inflation factor (VIF) was important to ensure the reliability of the regression model. VIF explains how much multicollinearity, which is the correlation between predictors, is present. A VIF under 5 was considered an acceptable multicollinearity.

## 3. Results

### 3.1. Age with PWVao, Skin SAF Value, and IMT

**Figure 1:**
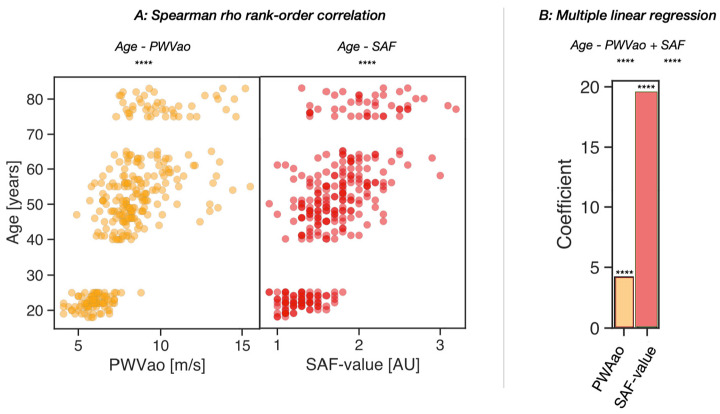
Associations between age and PWVao (yellow) and skin SAF value (red), respectively, in the cohort (*n* = 309). (**A**) The Spearman’s correlation between age with PWVao and skin SAF value is visualized in the scatter plot. (**B**) The bar plot reveals the contributions and the association significances of the independent variables, PWVao and SAF value, with the dependent variable, age, in a multiple linear regression model. Significances is given as ****: *p* ≤ 1.00 × 10^−4^.

First, we assessed the relationships between age, skin SAF value, and PWVao in a group of middle-aged individuals aged 40 to 65 years old (*n* = 309). Both parameters revealed highly significant correlations with age, with PWVao showing a correlation coefficient of 0.75 (*p* = 5.74 × 10^−58^) and age with SAF showing a correlation coefficient of 0.69 (*p* = 1.03 × 10^−45^) (Figure 1A). To determine the relative contributions of these parameters to age, we used a multiple regression model. We found that SAF had a higher regression coefficient than PWVao, with a coefficient of 19.52 (*p* = 9.18 × 10^−25^) compared with PWVao’s coefficient of 4.19 (*p* = 5.24 × 10^−28^) (Figure 1B).

**Figure 2:**
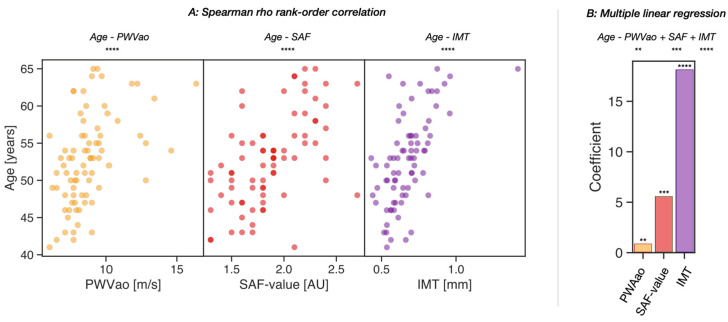
Association between age and the parameters of vascular function, PWVao (yellow), skin SAF value (red), and IMT (purple), in the subgroup of participants with all three measurements (*n* = 77). (**A**) Spearman’s correlations between age and the three variables are depicted in the scatter plot. (**B**) The bar plot reveals the contributions of the three independent variables PWVao, skin SAF value, and IMT, with the dependent variable, age, in a multiple linear regression model. Significances are given as **: 1.00 × 10^−3^ < *p* ≤ 1.00 × 10^−2^; ***: 1.00 × 10^−4^ < *p* ≤ 1.00 × 10^−3^; and ****: *p* ≤ 1.00 × 10^−4^.

Next, we analyzed the correlation between age and all three parameters of vascular function in a subset of the middle-aged group who also underwent IMT measurement (*n* = 77). We analyzed the Spearman’s rank correlation between the age of the individuals and the three parameters of vascular function (PWVao, skin SAF, and IMT). All of them showed highly significant correlations with age, with PWVao showing a correlation coefficient of 0.53 (*p* = 6.98 × 10^−7^), SAF showing a correlation coefficient of 0.58 (*p* = 2.66 × 10^−8^), and IMT showing a correlation coefficient of 0.63 (*p* = 7.89 × 10^−10^) (Figure 2A). Multiple linear regression models were applied to gain insight into the relative contributions of the three parameters with age. We found that IMT had the closest association with age, with a coefficient of 18.07 (*p* = 1.74 × 10^−6^), while SAF and PWVao showed weaker associations, with coefficients of 5.53 (*p* = 8.73 × 10^−4^) and 0.82 (*p* = 2.89 × 10^−3^), respectively (Figure 2B).

### 3.2. Interrelationship between Skin SAF Value and PWVao and IMT

**Figure 3:**
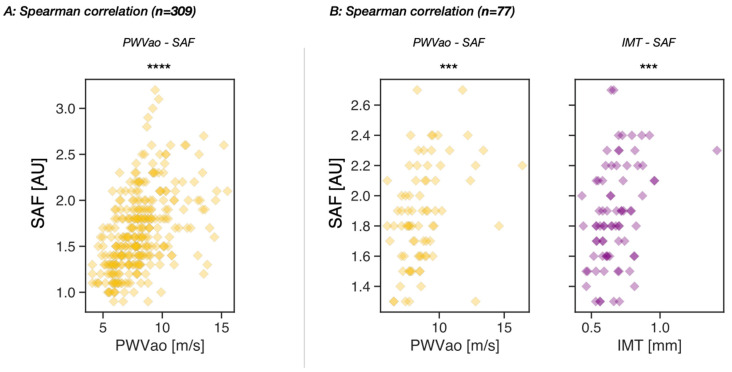
Interrelationship analysis between skin SAF values and PWVao or IMT. (**A**) Spearman’s correlation between PWVao and SAF values (yellow) in cohort (*n* = 309); (**B**) spearman’s correlation between PWVao (yellow) or IMT (purple) with SAF values in cohort (*n* = 77); Significances are given as ***: 1.00 × 10^−4^ < *p* ≤ 1.00 × 10^−3^; and ****: *p* ≤ 1.00 × 10^−4^.

The results of this study show a significant and positive association between SAF values and PWVao in the larger cohort of middle-agers consisting of 309 individuals, with a Spearman’s correlation coefficient of 0.54 and a *p*-value of 6.48 × 10^−25^ (Figure 3A). Age did not appear to play a moderator role in the relationship between the SAF value and PWVao across the middle-aged group, as the confidence interval of LLCI to ULCI crossed zero, as evidenced by Table 1.

In the smaller cohort (*n* = 77), the relationship between SAF values and IMT or PWVao was investigated. A positive and significant Spearman’s correlation in both pairs was confirmed with Spearman’s correlation coefficients of 0.39 and 0.40, respectively, and *p*-values of 4.16 × 10^−4^ and 3.43 × 10^−4^, respectively (Figure 3B). Again, no moderation effect of age was detected in the relationship between SAF values and IMT or PWVao since in this age range, the confidence intervals did contain zero, as shown in Table 2.

### 3.3. Relationship between Skin SAF Value and Other Parameters

We further analyzed how other phenotypic variables were correlated with skin SAF values in the three age groups of the cohort (*n* = 309) with a multiple linear regression analysis. As shown in Table 3, the VIF of each variable was below the value five. Apart from the information in the table, the regression model reported an F-value equal to 1.26 × 10^−36^.

**Figure 4:**
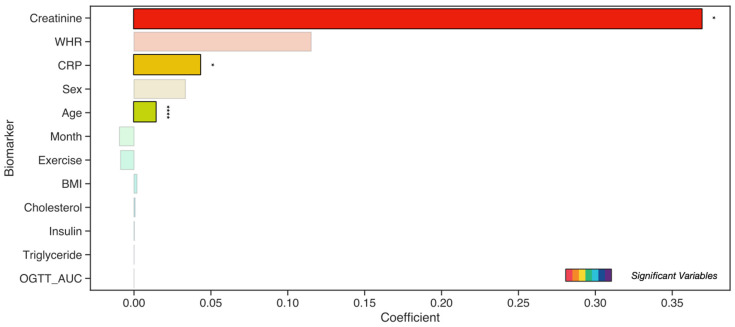
Multiple linear regression analysis for assessing the relationship between the skin SAF values and phenotypic factors in the total cohort (*n* = 309). The bar size represents the value of the corresponding coefficient of each independent variable. It shows their contribution to the relationship with the skin SAF value. Statistically significant coefficients are indicated by solid-colored bars, while translucent bars are non-significant. Significances are given as *: 1.00 × 10^−2^ < *p* ≤ 5.00 × 10^−2^; and ****: *p* ≤ 1.00 × 10^−4^.

It was observed that phenotypic variables such as age, creatinine, and CRP had a significant and positive association with SAF values after adjusting for the rest of the factors (Figure 4). To gain a better understanding of the relationship between the different variables in our dataset, we also examined their pairwise Spearman’s correlations, as shown in Supplementary Figure S1.

### 3.4. Seasonal Effect on Skin SAF Values

We also investigated whether there was a seasonal effect on skin SAF values. We acquired deeper insight into the skin SAF value difference between the summer (from May to September) and winter months (from October to April). The definition of summer and winter was based on information about sunshine hours and temperatures during the duration of this study provided by the German Weather Service (Supplementary Figure S2). However, after replacing the month variable with the season variable in our multiple linear regression model, we found no significant association between skin SAF values and season (*p*-value = 1.03 × 10^−1^).

## 4. Discussion

AGE products are powerful chemical structures conferring a significant risk for various diseases. Their pro-inflammatory properties have made the respective RAGE receptor a promising target, and various small molecules have been designed and tested to blunt disease progression [29]. In addition to this positive influence on the course of Alzheimer’s disease, metabolic complications could also be targeted in the future.

The deposition of these AGEs in the body makes them easily accessible by measuring their fluorescent properties in the skin. This measure was previously identified as a significant marker for cardiovascular disease [1]. According to our data, PWVao, which is another established marker of cardiovascular risk and prognosis [30,31], was significantly associated with SAF. Furthermore, SAF is significantly associated with intima–media thickness, which has also been tested as a surrogate marker for cardiovascular complications [32]. All these techniques are, therefore, helpful for the early diagnosis of a specific cardiovascular disease risk. Technically, the mortality risk due to PWVao alterations can be quantified with an increase of up to 39% for each 1 m/s increment [33]. However, for reproducible and reliable measures, the examination procedure has to be highly standardized to avoid incorrect measurements as some substances, e.g., alcohol, nicotine, or caffeine, interfere with the readings. Additionally, PWVao may be misinterpreted in certain pathological conditions such as heart failure with an ejection fraction of less than 50% [34]. Those patients were excluded from our study. IMT measurement using ultrasound, on the other hand, requires trained and experienced examiners to be able to interpret vascular physiology at the scanning site correctly. It also needs some technical prerequisites that might not be available in every study location.

In turn, SAF is considered to be the most straightforward and least expensive technique for measuring AGEs deposition. It can be measured within seconds and does not require any extensive training. However, there might be some restrictions in its application. One such restriction is that natural skin pigmentation can interfere with readings. According to Fitzpatrick et al., six skin types can be subdivided from ivory (I) to dark brown (VI) [35]. In our study, only Caucasians were selected, which generally only includes types I to IV. Although the skin types of the individual subjects were not assessed, we assumed an equal distribution across the types I–IV, as is usually observed in the Caucasian population. Additionally, tanning might interfere, as sun exposure has already been shown to affect SAF readings in other cohorts [36]. In our cohort, however, no seasonal impact could be demonstrated (Supplementary Figure S1), which agrees with other studies reporting that the seasonal impact on SAF values in human skin was limited among their control and diabetic patients [37,38].

SAF was significantly and positively associated with age. Although IMT was revealed to have the highest coefficient in the multilinear regression analysis (Figure 2), IMT, PWVao, and SAF were not measured on the same scale. For IMT, a change of one unit might be quite large, whereas a change of three units for SAF is relatively realistic. Thus, this study concluded that skin SAF value is of comparable value, while SAF measurement has the advantages of simplicity and short duration for measurement with high reproducibility [39].

Further analysis of the interrelationships revealed significant correlations between SAF and IMT and PWVao (Figure 3A,B). However, since the three parameters all correlate with age, it is thus essential to verify the independence of the valuated interrelationships. Based on a moderation analysis, we confirmed that the associations between skin SAF and IMT and PWVao were significantly independent of age in both cohorts (Table 1 and Table 2).

Comparing the combined effect of all tested variables in Table 1, an F-value equal to 1.26 × 10^−36^ indicated a significant performance in our regression model. Standard errors revealed that the covariance matrix of the errors was correctly specified. Meanwhile, every parameter had a variance inflation factor (VIF) under five, meaning that no severe or moderate multicollinearity should be considered in the model. Variables that shared higher VIF values, such as sex and WHR, implied a potentially strong correlation between those two parameters (Supplementary Figure S2).

Due to our multilinear regression model, creatinine levels were positively and significantly associated with skin SAF values, which is supported by other research works. Sharp et al. confirmed the significant correlation between serum creatinine and low-molecular-weight AGEs, which was measured using fluorescence spectroscopy, in both healthy non-diabetic volunteers (*n* = 106) and patients with diabetes (*n* = 499). Similarly, Stam and his team pointed out a significant relationship between AGE peptides and creatinine clearance [40,41].

Additionally, our study also found a significant positive relationship between SAF values and CRP levels. This can be explained, at least in part, by the stimulatory role of AGE on the expression of interleukin-6 (IL-6) and interleukin-1 beta (IL-1β) from monocytes, which in turn raises CRP production in the liver [42].

## 5. Conclusions

Being a fast, non-invasive, and reproducible way to estimate AGE deposition, SAF seems to be of comparable power for predicting cardiovascular risk to more elaborated technical tests. Moreover, SAF reveals positive correlations with multiple phenotypical parameters such as age, IMT, PWVao, CRP, and creatinine. Using SAF as a time-saving and simple screening method thus deserves more attention in the relevant clinical setting.

## Figures and Tables

**Table 1:** Conditional effects of PWVao on skin SAF at the values of the moderators in the cohort *n* = 309. Three representative age values were chosen. SE stands for standard error. T-value is abbreviated as T.

PWVao-SAF						
Age	Effect	SE	T	*p*-Value	LLCI	ULCI
2.85 × 10^1^	−3.40 × 10^−3^	1.78 × 10^−2^	−1.93 × 10^−1^	8.47 × 10^−1^	−3.83 × 10^−2^	3.14 × 10^−2^
4.75 × 10^1^	2.80 × 10^−3^	1.24 × 10^−2^	2.23 × 10^−1^	8.24 × 10^−1^	−2.16 × 10^−2^	2.71 × 10^−2^
6.65 × 10^1^	9.00 × 10^−3^	1.26 × 10^−2^	7.16 × 10^−1^	4.75 × 10^−1^	−1.56 × 10^−2^	3.36 × 10^−2^

**Table 2:** Conditional effects of IMT (**A**) or PWVao (**B**) on skin SAF at the values of the moderators in the cohort *n* = 77. Three representative age values were chosen. SE stands for standard error. T-value is abbreviated as T.

**A: IMT-SAF**						
**Age**	**Effect**	**SE**	**T**	***p*-Value**	**LLCI**	**ULCI**
4.67 × 10^1^	1.34 × 10^−1^	5.00 × 10^−1^	2.68 × 10^−1^	7.90 × 10^−1^	−8.47 × 10 ^−1^	1.11 × 10^0^
5.28 × 10^1^	1.21 × 10 ^−1^	3.48 × 10^−1^	3.48 × 10 ^−1^	7.29 × 10 ^−1^	−5.61 × 10 ^−1^	8.03 × 10^−1^
5.89 × 10 ^1^	1.08 × 10 ^−1^	2.78 × 10^−1^	3.88 × 10^−1^	6.99 × 10^−1^	−4.38 × 10^−1^	6.53 × 10^−1^
**B: PWVao-SAF**						
**Age**	**Effect**	**SE**	**T**	***p*-Value**	**LLCI**	**ULCI**
4.67 × 10^1^	−2.28 × 10^−2^	3.39 × 10^−2^	−6.73 × 10^−1^	5.03 × 10^−1^	−8.92 × 10^−2^	4.36 × 10^−2^
5.28 × 10^1^	5.80 × 10^−3^	2.14 × 10^−2^	2.71 × 10^−1^	7.87 × 10^−1^	−3.62 × 10^−2^	4.78 × 10^−2^
5.89 × 10^1^	3.44 × 10^−2^	2.03 × 10^−2^	1.69 × 10^0^	9.46 × 10^−2^	−5.40 × 10^−3^	7.42 × 10^−2^

**Table 3:** Multiple linear regression analysis for studying the relationship between SAF value with phenotypic factors. VIF presents the multicollinearity indicator of corresponding variables. Significant factors (*p* ≤ 5.00 × 10^−2^) are indicated by bold letters. SE stands for standard error. T-value is abbreviated as T.

Independent Variable	Coefficient	SE	T	*p*-Value	VIF
Age	1.42 × 10^−2^	1.00 × 10^−3^	1.07 × 10^1^	0.00 × 10^0^	1.91 × 10^0^
BMI	1.90 × 10^−3^	6.00 × 10^−3^	3.42 × 10^−1^	7.33 × 10^−1^	1.91 × 10^0^
Cholesterol	7.00 × 10^−4^	1.00 × 10^−3^	1.26 × 10^0^	2.09 × 10^−1^	1.61 × 10^0^
Const ^a^	5.05 × 10^−1^	3.20 × 10^−1^	1.58 × 10^0^	1.16 × 10^−1^	0.00 × 10^0^
Creatinine	3.69 × 10^−1^	1.59 × 10^−1^	2.31 × 10^0^	2.10 × 10^−2^	1.49 × 10^0^
CRP	4.29 × 10^−2^	1.80 × 10^−2^	2.33 × 10^0^	2.00 × 10^−2^	1.04 × 10^0^
Exercise ^b^	−8.70 × 10^−3^	1.40 × 10^−2^	−6.18 × 10^−1^	5.37 × 10^−1^	1.04 × 10^0^
Insulin	2.00 × 10^−4^	5.00 × 10^−3^	4.60 × 10^−2^	9.63 × 10^−1^	1.45 × 10^0^
Month	−9.50 × 10^−3^	5.00 × 10^−3^	−1.78 × 10^0^	7.60 × 10^−2^	1.02 × 10^0^
OGTT_AUC	−3.48 × 10^−6^	5.27 × 10^−6^	−6.59 × 10^−1^	5.10 × 10^−1^	1.30 × 10^0^
Sex ^b^	3.34 × 10^−2^	5.80 × 10^−2^	5.78 × 10^−1^	5.64 × 10^−1^	2.52 × 10^0^
Triglyceride	7.21 × 10^−5^	0.00 × 10^0^	1.80 × 10^−1^	8.58 × 10^−1^	1.48 × 10^0^
WHR	1.15 × 10^−1^	3.28 × 10^−1^	3.51 × 10^−1^	7.25 × 10^−1^	3.07 × 10^0^

^a^ The constant term ensures that the model will be unbiased; ^b^ Data was transformed into numerical status from original categorical data.

## Data Availability

The data presented in this study are available on request from the corresponding author. The data are not publicly available due to local data protection rules.

## Supplementary Materials

The following supporting information can be downloaded at: https://www.mdpi.com/article/10.3390/nu15071597/s1. Figure S1. Meteorological data of Weihenstephan-Dürnast, Bavaria Germany from January 2016 to December 2018. Figure S2. Spearman rho rank correlation of all variables visualized in a heat map.
