# Supplementary material for: Skin Autofluorescence Mirrors Surrogate Parameters of Vascular Aging: An Enable Study
Source: Nutrients. 2023 Mar 25;15(7):1597. doi: 10.3390/nu15071597 (PMC10096848; doi:10.3390/nu15071597)
Supplement: Supplementary file 1 [file nutrients-15-01597-s001.zip › nutrients-2199659-supplementary.pdf]

Supplementary Material

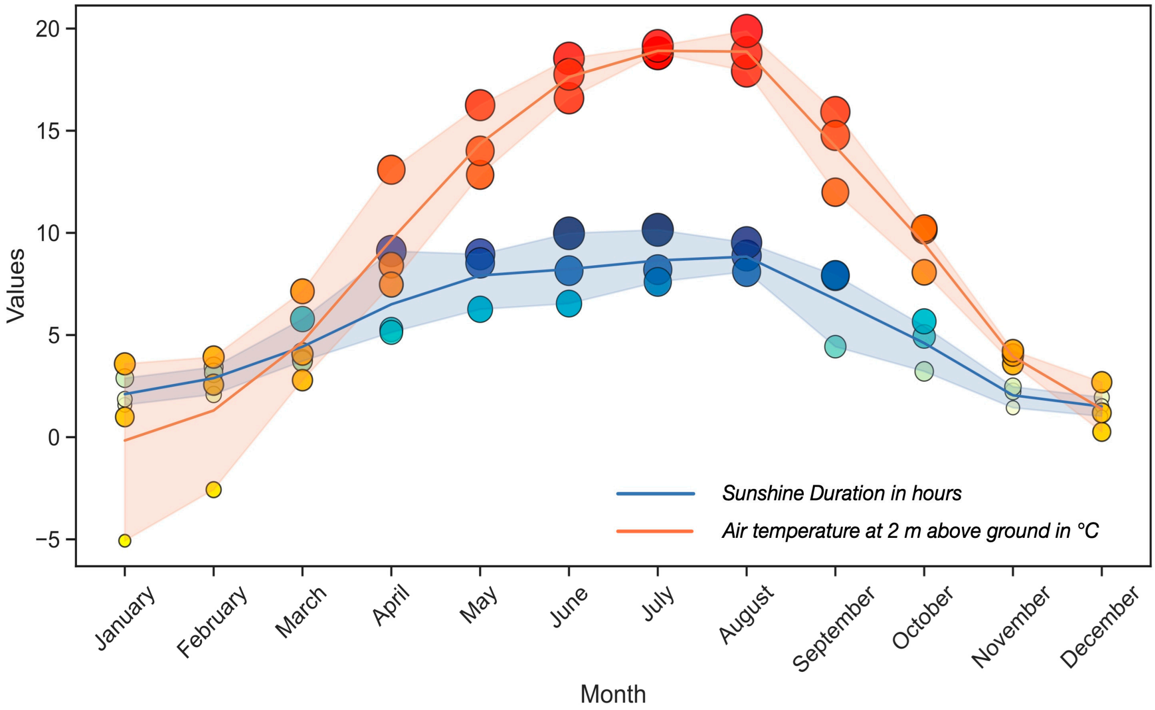

Supplement Figure S1. Meteorological data of Weihenstephan-Dürnast, Bavaria Germany from January 2016 to December 2018. Warm colored lines and circles presents monthly mean of station observations of air temperature at 2 m above ground in °C, whereas lines and circles with cool color indicates monthly mean of station observations of sunshine duration in hours. Circle size and color are adjusted upon its temperature and sunshine hours.

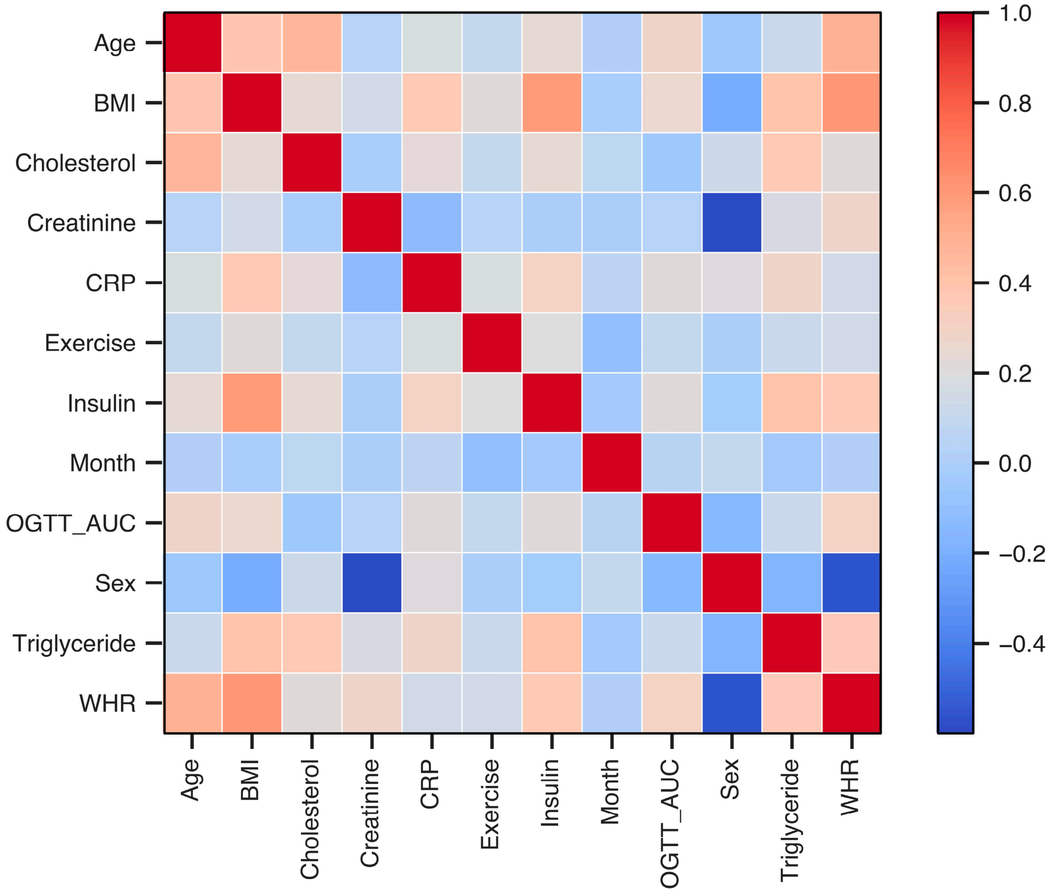

Supplement Figure S2. Spearman rho rank correlation of all variables visualized in a heat map. The color bar on the right defines positive and negative pairwise correlations in a cool and warm color. The deeper the color in a square, the stronger the correlation and vice versa.
